# Concerned or Apathetic? Using Social Media Platform (Twitter) to Gauge the Public Awareness about Wildlife Conservation: A Case Study of the Illegal Rhino Trade

**DOI:** 10.3390/ijerph19116869

**Published:** 2022-06-03

**Authors:** Siqing Shan, Xijie Ju, Yigang Wei, Xin Wen

**Affiliations:** 1 School of Economics and Management, Beihang University, Beijing 100191, China; shansiqing@buaa.edu.cn (S.S.); weiyg@buaa.edu.cn (Y.W.); wenxin_199511@buaa.edu.cn (X.W.); 2 Beijing Key Laboratory of Emergency Support Simulation Technologies for City Operation, Beijing 100191, China

**Keywords:** rhino trade, social media, text mining, sentiment analysis, data visualization

## Abstract

The illegal wildlife trade is resulting in worldwide biodiversity loss and species’ extinction. It should be exposed so that the problems of conservation caused by it can be highlighted and resolutions can be found. Social media is an effective method of information dissemination, providing a real-time, low-cost, and convenient platform for the public to release opinions on wildlife protection. This paper aims to explore the usage of social media in understanding public opinions toward conservation events, and illegal rhino trade is an example. This paper provides a framework for analyzing rhino protection issues by using Twitter. A total of 83,479 useful tweets and 33,336 pieces of users’ information were finally restored in our database after filtering out irrelevant tweets. With 2422 records of trade cases, this study builds up a rhino trade network based on social media data. The research shows important findings: (1) Tweeting behaviors are somewhat affected by the information of traditional mass media. (2) In general, countries and regions with strong negative sentiment tend to have high volume of rhino trade cases, but not all. (3) Social celebrities’ participation in activities arouses wide public concern, but the influence does not last for more than a month. NGOs, GOs, media, and individual enterprises are dominant in the dissemination of information about rhino trade. This study contributes in the following ways: First, this paper conducts research on public opinions toward wildlife conservation using natural language processing technique. Second, this paper offers advice to governments and conservationist organizations, helping them utilize social media for protecting wildlife.

## 1. Introduction

Illegal wildlife trade is a widespread and one of the most challenging issues for global biodiversity conservation and cooperation [1]. The world is facing a surge in illegal wildlife trade that poses a serious threat to biodiversity conservation [2]. Following narcotics, human trafficking, and counterfeiting, illegal wildlife trade is the fourth among the transnational organized crimes [3]. Billions of plants, animals, and related products are sold [4] as trophies, food, clothing, and medicine to satisfy human demands [5]. Although it is difficult to estimate the annual volume of wildlife trade between countries, it was reported in 2013 that its legal trade is worth USD 323 billion [6]. Such uncontrolled wildlife trade is not only a threat to the survival of species, but it also has a negative effect on society, such as the propagation of infectious diseases [7]. Furthermore, the severe impact of rampant trades in wildlife on ecosystems will eventually affect the lives of the current and future generations of human beings [8]. This is why more attention is being paid to wildlife trade.

The eager pursuit of rhino products leads to a soaring demand in the global wildlife market. For instance, rhino horn products are often regarded as a symbol of social status. They are made into cups, bowls, hairpins, and other decorative items and usually cost quite a bit [9]. In addition, rhino horns are believed to have medicinal properties and are used to cure fevers, heart diseases, food poisoning, and so on. However, it should be remembered that rhino horn has the same composition as human hair and fingernails [10]. These misconceptions increase the demand for rhinos in the world market.

**Figure 1:**
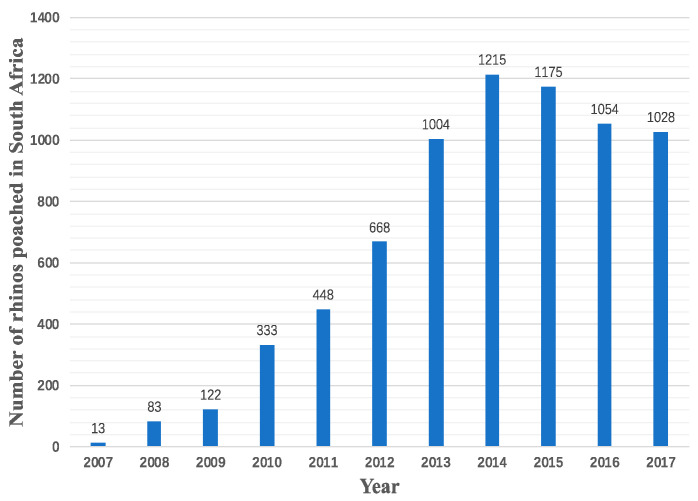
The number of rhinos poached in South Africa (2007–2017).

There are five rhino species distributed across Africa and Asia, including two African species (white rhino and black rhino) and three Asian species (Indian rhino, Sumatran rhino, and Javan rhino). They are facing a high possibility of extinction because of poaching and habitat loss. South Africa, which is home to the largest population of rhinos (nearly 80%), witnesses the greatest number of poaching cases. It is reported that rhino poaching in South Africa alone is up 9000%, from 2007 to 2014. Although there is a drop in the numbers since 2015, over 1000 rhinos continue to be a victim of poaching each year [11] (Figure 1).

Many international and local institutions have dedicated themselves to wildlife conservation. The Convention on International Trade in Endangered Species of Wild Fauna and Flora (CITES) is an international agreement that aims at guaranteeing the survival of wildlife despite trade [12]. The CITES Trade Database was erected by the UNEP World Conservation Monitoring Centre, which covers over 15 million (2015) cases of trade regarding living animals, plants, and their by-products [13]. This database contributes to monitoring illegal trade levels and provides a summary of the major trade between countries.

Apart from the traditional media, social media has provided us with an alternate way to learn about wildlife conservation. It has become relatively convenient to express concerns about wildlife conservation [14]. The rapid development of social media platforms has allowed people to share their opinions easily. Popular social media platforms such as Facebook, Twitter, and Instagram are being used as a source of information through techniques such as crowdsourcing [15]. Twitter dominates social media platforms worldwide, with up to 126 million monetizable day active users, according to the financial report for the fourth quarter of 2018 [16]. Millions of users receive and publish short messages limited to 280 characters called “tweets” every day. Social media has become an important role in social-related causes. Alnazzawi considers that Twitter can be used to detect hate crimes and their motivations [17]. Social media can help pastoralists make the decision on livestock mobility to face climate change and human mobility [18]. Hu et al. found that Twitter can improve inter-organizational emergency communication [19]. Social media can also be used for rapid emergency damage evaluations in real-time [20]. Environmental organizations regard Twitter as a channel to broadcast conservation-related information. From Twitter, we can collect and analyze public opinion on boycotting wildlife trade or appeal to governments to ban the trade [21]. Twitter has become a powerful tool to collect data and gain insight into how this issue is viewed among the masses. Thus, social media gives us a completely new perspective on analyzing rhino trade trends, which is quite different from using traditional data sources.

This study aims to highlight how social media information can be used for public opinion monitoring of social events. We follow the Agenda-setting theory to focus on the public reaction to rhino trade with overall trade worldwide as the backdrop [22]. To achieve the research objective, this study includes three questions: (1) Who plays an influential role when it comes to rhino trade? (2) What topics do the public pay more attention to when talking about rhino trade? (3) Where do the public’s attitudes become inclined in the process of communication?

The contributions and novelty of this study lie in the following two aspects. First, this paper provides a framework for analyzing rhino protection issues by using Twitter and following the Agenda-setting theory. Second, public sentiment about rhino trade is measured and geographic distribution features are discovered against the backdrop of global rhino trade situations.

This paper is structured as follows. Section 2 provides an extensive literature review. Section 3 introduces the data and methods. Section 4 discusses the research findings. Section 5 concludes the research outcomes, and policy recommendations are provided.

## 2. Literature Review

### 2.1. Traditional Research on Wildlife Conservation

The rampant illegal wildlife trade is deleterious for bioecology conservation. Researchers have attempted to analyze this trend through surveys on the wildlife trade globally. Ribeiro conducted a survey on bird conservation based on the consolidated resources of the CITES database and expert knowledge, and they inferred that socio-cultural motivations and demand for wild pet-bird may result in legal bird trade cases [23]. Veríssimo and Wan focused on the issue of how to reduce human demand for wildlife by surveying literature on demand reduction actions [24]. Research also concentrates on region-specific wildlife trade. For example, by using semi-structured interviews, Elizabeth investigated the pet trade for domestic markets in Peru and surveyed the role of NGOs in wildlife conversation [25]. Nijman investigated about the protection of marine mollusks in Indonesia from the perspective of laws and international agreements such as CITES. He also emphasized the importance of legislations and regulations, trade monitoring and illegal trade prosecutions, and wildlife protection [26].

The CITES Trade Database is widely used as an illegal wildlife trade database in the literature. Harfoot extensively study the scale, routes, and sources of wildlife trade based on CITES trade data from 1975 to 2014 for 28,282 species [27]. Symes proposed a gravity-underreporting modelling framework, with which they identified some drivers of wildlife trade and provided a quantitative assessment of trade flows [28]. However, Berec pointed out that certain limitations affect the availability of the CITES Trade Database and put forward several suggestions to improve it [29].

### 2.2. Wildlife Conservation Based on Social Media

Paudel recommend that there are many deficiencies in traditional media reporting [30]. Social media platforms are being used as a data source to study wildlife conservation. Di Minin reviewed the role of social media in conservation science and practice [31]. They suggested that spatio-temporal research on conservation can use social media data fully. Toivonen gave an overview of the methods used in studying conservation science through social media data [32].

A part of the literature also focuses on data mining methods for collecting, cleaning, and categorizing useful information on illegal wildlife trade from social media data. Social media platforms make it possible for wildlife dealers to release advertisements in the form of photos or texts [5]. Data mining technique is used to detect potentially illegal online sales of elephant ivory on eBay, and the detection rate accuracy is up to 93% [3]. A three-stage framework (mining, filtering, identifying) by using machine learning has been put forward to obtain illegal wildlife trade information buried in social media [33].

From social media, wildlife trade information can be monitored and evaluated through the analysis of the data on those platforms. Researchers monitor Facebook to obtain an overview of Asian otters’ species trade facts [34]. Integrating data from social media and other information sources along with expert knowledge gives researchers an insight on the scale and scope of wild-sourced grey parrot trade [35].

Social media can also contribute to drawing public attention to saving wildlife. The behaviors against wildlife conservation exposed on social media platforms, such as Facebook, can arouse great public attention and influence the government’s decisions regarding certain cases of illegal wildlife trade [36]. Social media, such as WeChat in China, are used to strengthen public awareness about wildlife conservation [8]. Features of popular threatened species on Twitter are discovered and used to increase the appeal of unpopular ones so that the public pays more attention to them [21].

### 2.3. Rhino Protection

Regarding literature on rhino protection, several researchers have focused on discovering trade network traits through official statistics. Such data are usually provided by traditional media, the CITES database, or other institutions dedicated to wildlife protection. Research based on traditional media, such as newspapers, reveals that the rhino horn trade volume in Chinese art and antiques markets has a significant correlation with poaching in South Africa [9]. The database of HealthMap Wildlife Trade was used to put forward a quantitative method to find key nodes in the illegal rhino trade network; the results found China, Mozambique, South Africa, Thailand, the United Kingdom, and Vietnam to be the IP fragmentation nodes [37]. SANParks database combined with data of the Asian population help researchers generate a model for evaluating the impact of various management policies on rhino sustainability [38].

Since rhinos are suffering from humans’ poaching activities, researchers are also interested in the motivations and preventions of poaching. Vu et al examined whether a legal rhino horn trade can reduce poaching through a choice experiment [39]. Ayling considered the internal and external sources of the network resilience of illegal rhino horns for reducing the trade [40]. Hubschle stated that the socio-political and historical context and continued marginalization of local people may be drivers of poaching [41]. Confronted with the rhino horn crisis, Crookes and Blignaut illustrated the importance of transformation from a sectoral conservation model to a more sustainable method that comprises a system dynamics model including five factors: rhino demand, rhino abundance, a price model, an income model, and a supply model [42]. In 2017, Crookes made further effort to build up a hybrid dynamical model [43]. He found that the reduction in rhino horn price would not be an effective way to prevent poaching; if poaching costs are increased simultaneously, the situation can be alleviated. However, the poachers’ costs, which are not in the policymaker’s control, made it difficult to stop poaching.

Although there are various studies based on social media data that discuss the threats faced by general or species-specific wildlife, very little research about rhino trade on Twitter has studied the relationship between social media content and news media [22] and how we can use social media to protect the rhino. Users on Twitter act as human sensors and contribute to natural sciences and conservation, thereby drawing public support from social media to arouse awareness about ecological protection. Therefore, this study follows Agenda-setting theory by throwing light on the discussion of rhino trade through Twitter to discover the discourse pattern of the public, including key topics discussed in the tweets, and excavates influential users and attitudes toward the issue. We believe that Twitter users’ behaviors will truly affect the public awareness of rhino protection.

## 3. Data and Methods

**Figure 2:**
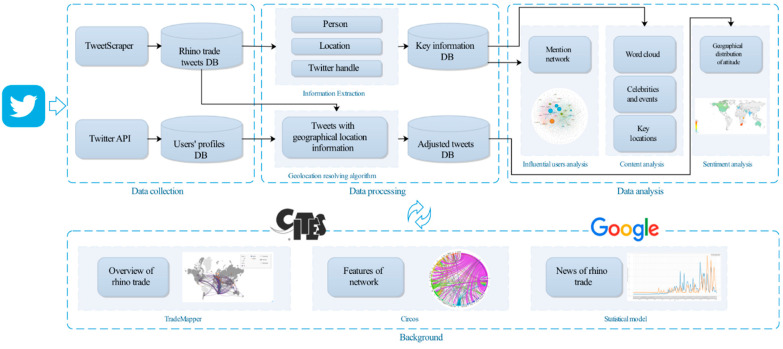
The framework of research.

The framework of our study is shown in Figure 2. This study collected trade data from the CITES Trade Database and related online news from Google and conducted an analysis to evaluate the global rhino trade situation. Then, we used social media data to learn about the public discourse on this topic. First, tweets and users’ profiles were collected by TweetScraper [44] and Twitter API. Second, based on Twitter information, we extracted useful contents and resolved location information in the data-processing phase. Specifically, we discovered influential users, analyzed tweets’ contents, and studied the public’s attitude.

### 3.1. Data

#### 3.1.1. Background Data

The data regarding rhino’s global illegal trade are sourced from the CITES Trade Database [13]. Each trade record has details about a shipment between two countries, including basic information about the species traded, the importer, the exporter, the origin, the reported quantity, and the term, unit, purpose, and source [27]. Data on the trade in Rhinocerotidae for all purposes from 2007 to 2017 include 2422 records that are accessed from the CITES Trade Database.

To learn about rhino trade discussion in the traditional mass media, this paper also accessed online information by crawling Google news with the keywords rhino trade. Finally, 176 news pieces were gathered from 2007 to 2017.

#### 3.1.2. Twitter and Users’ Profiles

Rhino-trade-related tweets were collected from Twitter by using Python. Since Twitter API only allows us to obtain the past seven days’ data [45], in order to obtain more historical data, we use a simple spider called TweetScraper for data collection. This study set “rhino trade” as the keyword to grab data from Twitter, and the time period was set as 1 January 2007 to 31 December 2017. After filtering out irrelevant tweets, 83,479 useful tweets were finally collected into our database for analysis.

For further analysis, we needed more information on users who have tweeted posts about the rhino trade during the time range that we grabbed data. Users’ information can be crawled through their ID, which we can obtain from the previous step. After collection, 33,336 pieces of users’ information were obtained.

### 3.2. Methods

#### 3.2.1. Building up Rhino Trade Network

The data sourced from CITES record details about a trading case, including importer, exporter, origin, reported quantity, unit, term, purpose, etc. For different terms, the reported quantity is recorded using a different unit. When trading routines were analyzed, the specific trading volume was ignored, and only one trading case to a trading volume of 1 was simplified to unify the data. That is, only the number of trading cases between different countries was considered. In each record, the exporter and origin may be two different countries. However, the origin country was used to analyze rhino trade routines across the world. Records with no accurate exporter or importer country code (“XX” or “XV” in the field) were deleted in the preprocessing phase.

Map-based trade monitoring is conducive to comprehending the worldwide dilemma of rhino trade. TradeMapper is a tool developed by TRAFFIC (a leading non-governmental organization working globally on wild animals and plants trade with respect to biodiversity conservation and sustainable development) and WWF-UK (the world’s leading independent conservation organization) [46]. This study used the tool to visualize our data from CITES to have a better understanding of the spatial patterns of trade.

This study also used Circos to make chord diagrams for gaining a different perspective on the trade data. Trade amounts and directions between countries can be clearly seen from the arcs.

#### 3.2.2. Information Extraction of Tweets Contents

One Twitter post usually contains information that may not be necessary for our analysis, such as Uniform Resource Locators (URLs), foreign language words, abbreviations, and symbols [45]. In order to remove these useless characters from our database, we use a tool package named ark-tweet-nlp. After removal, we hadcollected some useful information from Twitter posts in our database, such as Twitter handles, usernames, and locations.

**Twitter handle:** The Twitter handle represents a personal ID on Twitter, which starts with the “@” symbol. When users want to interact (direct mentions, retweets, and replies) with other users, they use Twitter handles in their posts. This study used the tool package named ark-tweet-nlp to tag words in each post and extract the information mentioned. After that, the poster ID and handles of a post will be recorded. This means, that mentioner (mentioning user)–mentionee (mentioned user) pairs will be obtained [47]. We can build up a directed relational network of users from the mentioner–mentionee pairs for further analysis.

**Location and username:** For discussing the key information in the tweets, this study used Named Entity Recognition (NER) to effectively extract the geographical and user name information involved in the tweets. NER is a task in the field of natural language processing. Important information, such as people, organizations, and localizations, as well as numerical expressions, can be extracted with NER [48].

#### 3.2.3. Geolocation Resolving Algorithm

Furthermore, in our study, it is necessary to identify the geographical origin of a Tweet. Adding a general location label or GPS coordinates to a Tweet are two ways to indicate the current location [49]. Due to the twitterers’ increasing awareness of privacy protection, they rarely include geographical information in tweets. For instance, more than 19 billion tweets were collected in a study conducted by Graham and his team, but less than 1% of the data contained geolocational information [50]. Thus, this study used a geolocation resolving algorithm, which makes use of locational data in users’ profiles [51]. Specifically, this study manually deleted invalid locational information only reserving locations with the granularity of a country. Invalid locational information may involve: (1) the user leaves the field “location” blank in the profile, (2) the content in the field “location” makes no sense or it has no relation to geographical information, (3) the content is only within the accuracy of the continent, (4) the content is ambiguous, or (5) the content is not in English.

After removing the invalid data, there remains 21,035 users’ profiles with location information that can be used in this research. Thus, the tweets collected can be easily defined with locations by combining them with profiles through the users’ ID number. This study eventually obtained 53,675 tweets with valid locations that can be used to analyze the geographical location information of twitterers.

#### 3.2.4. Influential Users’ Network

Gephi was used to draw a mention-relationship-directed graph, transferring a mentioner–mentionee pair into a directed edge from mentionee to mentioner. Based on the graph, the features of the network were figured out. The nodes whose outdegreewas is over 100 were listed out. Such users were classified into four types (media, non-governmental organization, governmental organization, and private company) according to their brief introduction on Twitter and search results on Wikipedia.

#### 3.2.5. Topic Analysis through Word Cloud

To analyze the topics of discourse, this study used Python to plot word clouds from 2009 to 2017 (only 3 tweets about rhino trade were posted in 2007 and 2008), where high-frequency words need more discussion.

#### 3.2.6. Geographical Distribution of Sentiment

Social media data can be used for sentiment analysis [52]. For the content of tweets that are separated, the lexicon-based method was utilized to analyze the emotional tendency of tweets. Terms on the Internet are different from those used in daily life, which means that specific Tweeting lexicon is necessary for this research. Therefore, the AFINN lexicon, which mainly contains words in the context of social media (Twitter), is used in this research. The latest version includes 2477 words, and each has been scored by a value of sentiment strength from −5 to +5 [53]. The public generally holds opposing attitude towards rhino-trade-related issues. In this study, we focus on the negative emotional index of tweets. Key indicators are defined as follows:

$$Neg-index=\frac{Nw}{Tw}$$

where Nw is the number of negative words in a Tweet, and Tw is the number of total words in a Tweet. The negative emotional index is always non-negative, and the higher the index is, the stronger the negative emotion is.

After calculating the negative emotional index of each tweet, country-wise values were summed up through the tweets’ locations obtained by the geolocation resolving algorithm mentioned previously, and the aggregated negative emotional index of each country was attained.

The usage of the Internet is found to vary among countries. To remove the effects of differences in figuring negative emotional index, this study used *Individuals using the Internet (%)* and *population (million)* data from The World Bank to normalize our calculation [54]. The steps taken to eliminate the differences in the year of examination were as follows:

1. Obtain a set of aggregated negative emotional indices $\left\{I_{i}|i\in C\right\}$, where $I_{i}$ denotes the aggregated negative emotional index of country i, and C denotes the set of countries that have aggregated negative emotional index.
2. Obtain $\left\{p_{i}|i\in C\right\}$ and $\left\{w_{i}|i\in C\right\}$ denoting the set *population* and *Individuals using the Internet* of country i, respectively, and then calculate $\left\{n_{i}=\frac{p_{i}w_{i}}{100}|i\in C\right\}$, which represents the population of Internet users in country i.
3. Use the max–min method to normalize the set of aggregated negative emotional index and the set of the population of Internet users. Thus, obtain two normalized sets, marking them as $\left\{{I\prime }_{i}|i\in C\right\}$ and $\left\{{n\prime }_{i}|i\in C\right\}$.
4. Find out the adjusted aggregated negative emotional index computed as $adjI_{i}=\frac{{I^{\prime }}_{i}}{{n^{\prime }}_{i}+0.01}$. (In case the denominator equals 0, add 0.01 to the denominator to make the fraction meaningful.)
5. Again, use the max–min method to normalize the set of adjusted indices and obtain $\left\{adjI_{i}\prime |i\in C\right\}$.

Finally, this study uses $\left\{adjI_{i}\prime |i\in C\right\}$ through Echarts to visualize the geographical distribution of the public attitude towards rhino trade.

## 4. Empirical Results and Discussion

### 4.1. Overall Level of Global Rhino Trade

From 2007 to 2017, this study obtained 2422 records of trade cases. There are 12 alternative purpose codes, from which this study selected hunting trophy (“H”), commercial (“T”), and personal (“P”) as illegal; 1538 records met the criterion.

**Figure 3:**
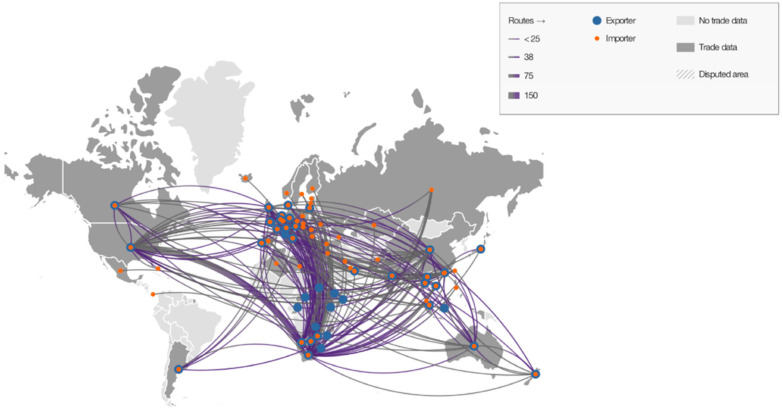
Aggregated routes of rhino trade cases, 2007–2017 (The CITES Trade Database).

From the annual global rhino trade situation (Figure 1) and the aggregated routines in Figure 3, it is clear that the African region is a major outflow region, and African rhinoceroses and their products are being shipped to North America, Europe, Asia, Oceania, and South America. The scope of trade is so wide that it involves nearly one-third of countries and regions worldwide (74 countries).

**Figure 4:**
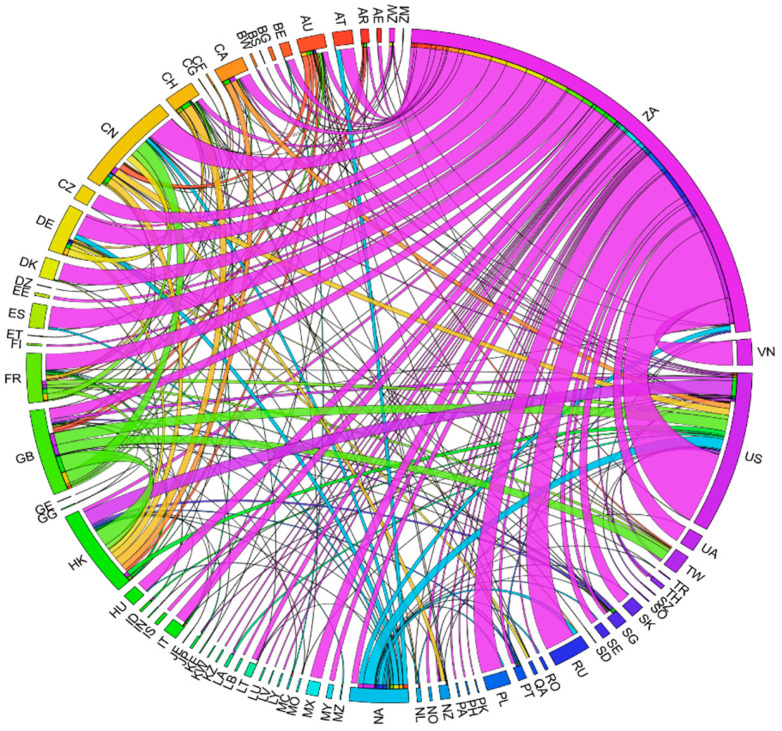
Routes of rhino trade cases, 2007–2017.

From the Circos results in Figure 4, we can intuitively notice the focus of rhino trade. South Africa is no doubt the biggest exporter, followed by the United Kingdom and Namibia, while China (China mainland and Hong Kong) is the largest importer, followed by the United States.

This study classified each trade case into two types: one is obtained from the wild source (Source code “W”), and the remaining cases are from other sources; 970 (63%) cases have been reported from the wild source. Figure 2 shows the steady growth in the annual ratio of wild sources to other sources.

Regarding wild-sourced rhinos for commercial, hunting, or personal purposes, 970 trade cases are reported from 13 countries and imports occur into 55. A total of 857 (88%) reported cases are from South Africa. The wild-sourced rhino and its products are transported mainly to the United States, China, Russia, and Vietnam, as shown in Table 1 and Table 2.

### 4.2. Effects of Online News on Tweeting

**Figure 5:**
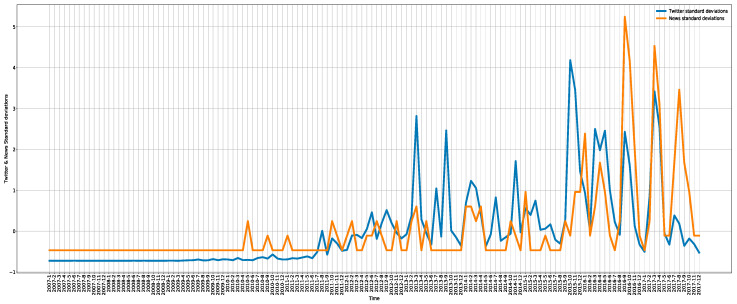
Detrended normalized monthly number of tweets and monthly number of online news on rhino trade.

At first, this study examined the effects of online news on tweeting behaviors. From the perspective of the numbers of tweets, as shown in Figure 5 these variables have a significant positive correlation (Pearson = 0.59, *p* < 0.001), and the trends of tweets and related news are highly consistent; the occurrences of major spikes tend to coincide.

To investigate and analyze the relationship between news and tweeting behaviors, a statistical model is used. When this study first uses the basic model, it shows a high level of autocorrelation (Durbin–Watson = 0.91). Then, the following models are constructed by applying first-differencing to convert the number of news and tweets into news and tweets standard deviations, respectively. With the transformed variables, the autocorrelation effects were virtually lowered (adj. R^2^ = 0.23, *p* < 0.001, Durbin–Watson = 2.51):

dTweets=A+βdOnlineNews+ε

### 4.3. Influential Users’ Analysis

When an “@” symbol was found in a Tweet, we recognized it as an approach to engage other users in the current conversation (both responding to other tweets and retweeting) [55].

**Figure 6:**
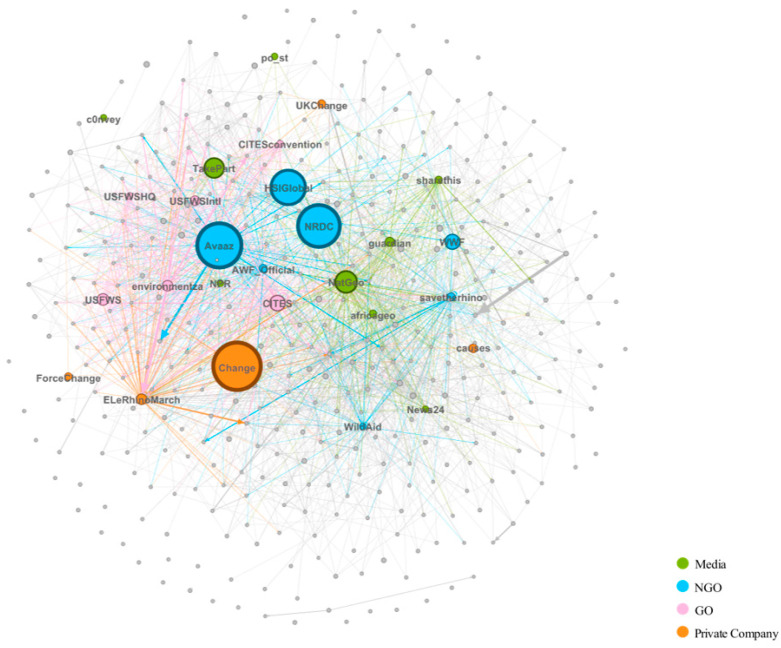
A directed graph of the mentioner–mentionee network.

This study found that several vital users are included in others’ tweets. In order to analyze the mentioner–mentionee network, this study built up a directed graph of the mentioner–mentionee network. As shown in Figure 6, this study obtained a total of 18,044 nodes in the diagram, and some of them have been filtered out in the process of visualization. Among these, 13,429 (74.4%) nodes’ outdegrees are 0, which means the users are not mentioned; rather, they follow others. In this study, users who were mentioned over 100 times were selected, and finally, the 27 top users were picked out. The top 27 users dominate because they were mentioned 11,584 times out of 23,994 (48.3%).

In the discourse of rhino trade, ordinary users were passionate about institutional users, which means that these Twitter accounts represent institutions. This study classified institutional users into four types according to a brief introduction in their Twitter accounts and Wikipedia, including news media, non-government organizations, government organizations, and private companies. The profiles of top users are shown in Table 3 below. The aggregated information of top users is shown in Table 4. Although the number of media users are highest among the 27 users, its contribution to their outdegrees is not remarkable. In contrast, NGOs have the most total outdegree followed by private companies. The performance of GOs in the top 27 users is not as remarkable as the other three types.

Those NGOs have extensive public foundations, and they contribute to protecting animals, specifically focusing on saving rhinos. Avaaz.org, an NGO, is a global civic movement connecting over 50 million citizens worldwide. They play a significant role in bringing people-powered politics to make the necessary decisions. NRDC (Natural Resources Defense Council) combines over 3 million members and online activists across the globe to protect the people’s rights to the air, water, and wild [56].

In addition, few individually owned companies or websites play an important role in discussing the rhino trade issue. They are usually platforms for petitions and may help in organizing campaigns around issues that impact the public. Change.org is the world’s leading civic organizing hub, empowering people everywhere to create the change they want to see, and it ranks at the top from the measurement of the outdegree.

### 4.4. Content Analysis

#### 4.4.1. Topics

**Figure 7:**
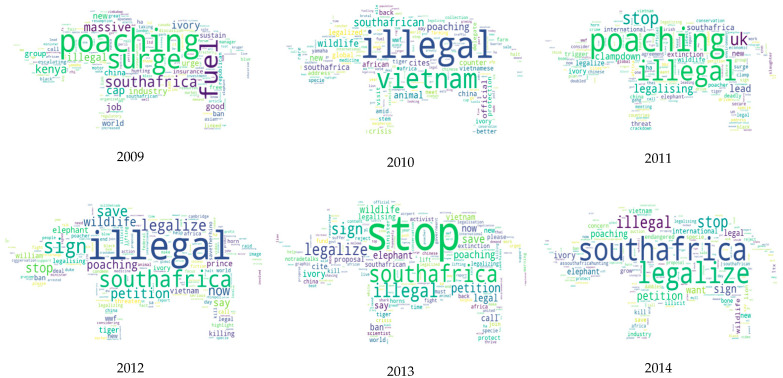

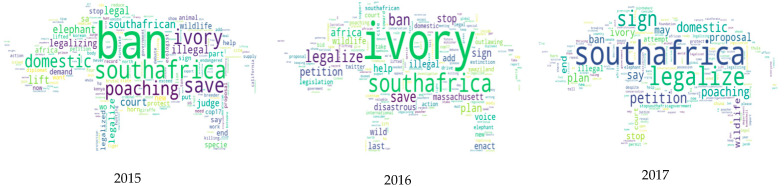
Word cloud of tweets each year from 2009 to 2017.

This study has drawn a word cloud of Twitter content for 2009–2017, as shown in Figure 7 below. From the high-frequency vocabulary that emerged from the discussions, the changes in the past decade can be seen clearly. The main discussion in 2009 was that “the surge of poaching” was mainly concentrated in African countries, including South Africa and Kenya. This study examined the tweets released in 2009 by keywords, discovering that users retweet the following: “Trade in rhino horn fuels massive poaching surge in South Africa” and “Kenya urges the world to sustain ban on trade in ivory, rhino horns”. In 2010, tweets focused on “South African delegates visit Vietnam to address illegal rhino horn trade”. “Illegal poaching” continued to be the main topic in 2011, and the UK was also receiving much attention: for instance, “UK leads clampdown on rhino horn trade”. In the tweets of 2011, discussions on the event of “SA considers legalizing rhino horn” can be found. Since 2012, Twitter has shown an explosive growth in public attention to “South Africa”, involving discussions about being “legal” or “illegal”. Additionally, there are several Tweeting vocabularies about “petition”, “stop”, and other strong emotions. There are retweets focused on “Stop South Africa from legalizing rhino horn trade” and “Don’t legalize the rhino horn trade”. Although the South African court has lifted the ban on domestic rhino horn trade, most conservationists and public are worried that legalizing this trade will lead to an increase in poaching.

#### 4.4.2. Locations

**Figure 8:**
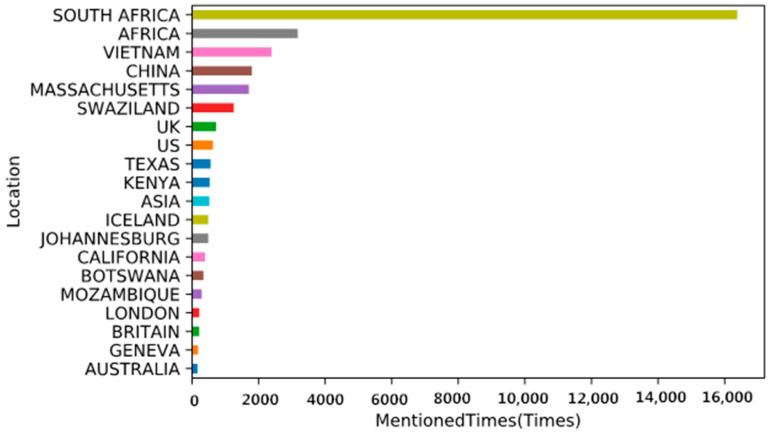
Location information mentioned in tweets.

This study used NER analysis to find the locations mentioned the greatest number of times on Twitter. Figure 8 shows the top 20 locations. South Africa is the focus in these tweets, including its biggest city, Johannesburg. Other countries in Africa are in second place. Countries in Africa are mentioned several times, including Swaziland, Kenya, Botswana, and Mozambique. South Africa is the home of rhinos, holding nearly 80% of the species. The surge of illegal rhino trade in Africa, particularly poaching, threatens the rhino population. Significantly, many suspected poachers are from Mozambique, some of whom are residents of Mozambique’s Limpopo National Park [57]. In addition, Kenya also suffers from heavy annual rhino loss due to poaching. Since the 1970s, the number of Kenya’s rhinos has decreased drastically, and human activities have left very little space for the species to recover [58]. Poaching and slaughtering have not stopped over the years. Thus, Kenyans are calling out to protect their rhinos. In contrast, Africans did not expect Botswana to contribute to rhino protection. However, it successfully replenished its rhino population after wildlife tourism development had almost resulted in the extinction of the species. Consequently, people have highlighted Botswana and its conservation methods. The Kingdom of Swaziland proposed to legalize rhino horn trade at the CITES Conference of Parties in 2016 [59], which caused extensive discussion on Twitter for some time.

Countries in Asia, including Vietnam and China, are among the top picks. Vietnam is a principal destination and transit country for illegal African rhino horn [60], while China is a big consumer of these products. These two countries have a large demand for rhino products because the Vietnamese and Chinese hold the widespread belief that rhino horns can be made into valuable medicine, which can even cure cancer [9].

In addition, the United States is also a focus country in this context, with Massachusetts ranking fifth, and other states, such as Texas and California, have been mentioned several times. The most cited European country is the United Kingdom, which is mentioned in various forms such as the UK, Britain, or London. Furthermore, Iceland, Geneva, and Australia are also on the list.

#### 4.4.3. Persons

**Figure 9:**
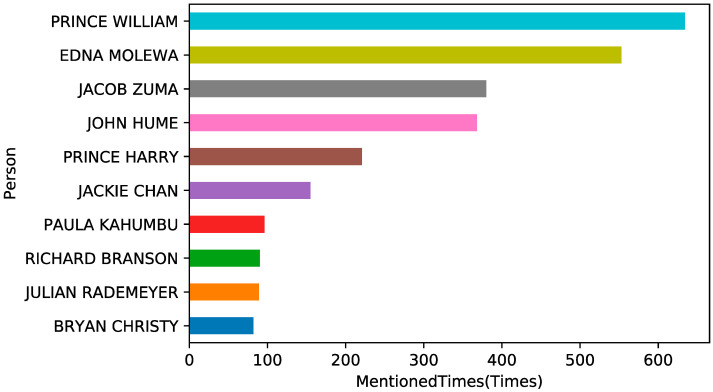
Information of people mentioned in tweets.

This study also uses NER to extract information on the names of the people involved in the tweets. It can be seen in Figure 9 that there are many celebrities across the world speaking out about rhino trade, including Prince William of the United Kingdom, Jacob Zuma of South Africa, the private owner of rhinos John Hume, Environmental Affairs Minister Edna Molewa, Chinese actor Jackie Chan, the Executive Director of WildlifeDirect Dr. Paula Kahumbu, and so on.

**Figure 10:**
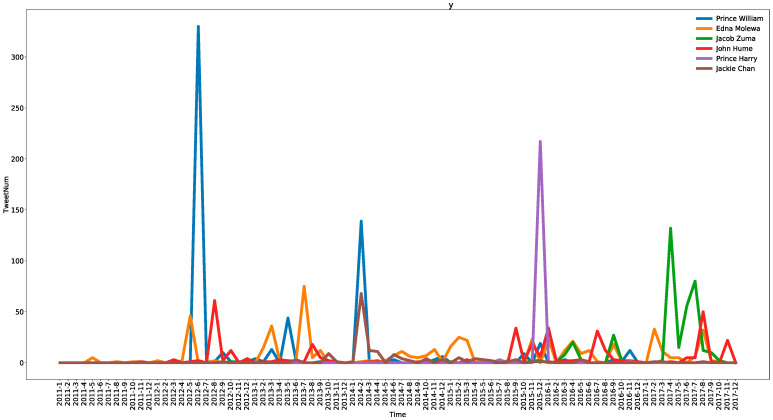
Trends of tweets related to celebrities over time.

Figure 10 shows tweeting trends related to the celebrities over time (the number of tweets over 100 in total). There is the greatest number of tweets related to Prince William, the Duke of Cambridge.

From Figure 10, we can observe that there are two spikes in tweets about Prince William. The first one occurs in June 2012 because he condemned rhino poachers and called for action to stop the illegal trade of rhinos. This and the tweets related to it take up half of the total, which can be discovered by the contents of tweets. Afterwards, Prince William and other royal family members, such as Prince Charles and Kate Middleton, helped in protecting rhinos, worked to highlight illegal wildlife trade, and aided the fight against wild trade; thus, the discussion on Twitter peaked in February 2014. Prince Harry was mentioned for the same reasons as Prince William. Tweets about him broke out in December 2015. Prince Harry paid a visit to South Africa and warned about the dangers of lifting a rhino horn trade ban, which drew the public’s attention.

There were over 500 tweets about Edna Molewa, the Minister of Environmental Affairs of South Africa. It is clear that these tweets have no outstanding points. However, they have an average distribution over time. Similarly, John Hume, the biggest rhino breeder in South Africa, contributes frequently to rhino protection activities.

Jacob Zuma is a South African politician who served as the fourth President of South Africa from the 2009 general elections until his resignation on February 14, 2018. He started to become concerned over the rhino trade sometime around 2016, according to Twitter. Moreover, in April 2017 and July 2017, the tweets related to his call to END RHINO HORN TRADE were retweeted widely, occupying over 50% of the total related tweets.

Another celebrity is Chinese action star Jackie Chan. “Tools of the Trade”, coproduced with the African Wildlife Foundation (AWF), features him in February 2014 [61]. This point and one of the spikes related to Prince William occur simultaneously because they include two new messages as a part of the world’s largest campaign to reduce the demand for endangered species products: “Whole World” and “Tools of the Trade”. However, in this study, it can be found that Prince William aroused more extensive attention on Twitter, and he had nearly two times the number of related posts than Jackie Chan. Undoubtedly, there are other celebrities featuring in the campaign, and their names can also be seen on Twitter.

From the content of the tweets, it is clear that the heads of state and social celebrities have paid close attention to this issue and attracted public attention. When they attend important events on wildlife trade, it attracts huge social attention for a certain period. The celebrities from the United States, the United Kingdom, and South Africa participate in events regarding rhino trade, which appeal to the public and motivate their awareness efforts about animal conservation. Online media platforms, such as news portals, also have the opportunity to publicize the details of such events on Twitter or other social media platforms. Other countries that also witnessed a high volume of trade either have few celebrities paying attention to rhino trade, or their activities are not influential enough. Celebrities may be significant contributors to wildlife conservation; moreover, they have a responsibility to do so.

### 4.5. Sentiment Analysis

In this study, we received a total of 53,675 tweets containing geographic information for subsequent analysis of the geographic distribution of public sentiment. Over 30,000 of the remaining tweets were discarded because the post user did not fill in the location or we were unable to effectively determine the location based on the content.

**Figure 11:**
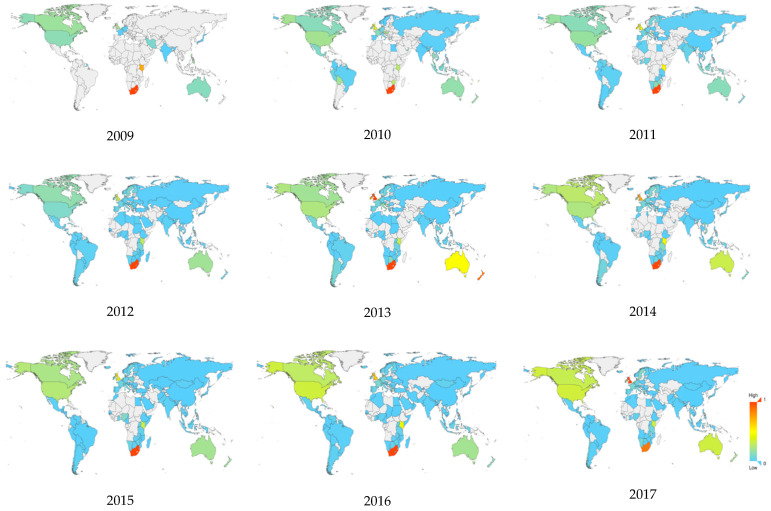
Geographical distribution of negative emotional index.

This paper plotted the adjusted negative public sentiment index (ranging from 0 to 1) of the 2009–2017 global public discussions on the rhino trade, as shown in Figure 11 it can be seen that the number of countries that have shown concern about this issue in tweets typically increases every year, with there being only 14 in 2009. By 2017, users of more than 100 countries had tweeted about the rhino trade. It is gratifying that the issue has aroused a worldwide concern. In addition, the maximum value of the negative sentiment index has changed slightly in the past decade. The maximum has always been in South Africa, indicating that the South African people are more concerned about this issue, and there are more manifestations in the tweets showing strong negative emotions. Following South Africa are Kenya and the United Kingdom (Table 5). Over 98% of Africa’s remaining rhinos are found in just four range states: South Africa, Namibia, Kenya, and Zimbabwe (A Traffic report). The reason that people react so strongly is that Africa, especially South Africa, is the country with the largest net outflow of rhinos. Moreover, South Africa’s legalizing rhino horn trade leads to an increase in the public’s concerns about rhinos and the subsequent opposition to the policy. Kenya, is home to around 1000 rhinos, two-thirds of which are critically endangered black rhinos [62]. Although there are few trade cases that are reported by CITES, its rhino poaching crisis is severe. The United Kingdom also has a significant illegal trade network with a large net outflow [37].

This study shows that Twitter users in South Africa have the strongest emotion most of the time, and the United Kingdom witnesses the strongest negative opinions in 2013 and 2017. The rest of the countries, such as most Asian, South African, and European countries, show concern about the affairs of rhino trade but do not display any noteworthy incident of negative opinions. Many African countries, however, pay little attention to this issue, even though they suffer from more loss of rhinos than the others. The Asian countries, such as Vietnam and China, which are considered to have the highest demand for rhino products, do not show much negative emotion toward the affair.

This study analyzed tweets on rhino trade issues in the background of the global overall trade situation. Online news that report relevant events are found to have a significant relationship with tweeting behaviors, which indicates that traditional media, such as official news, influence public opinion on social media to some extent. This result verifies the Agenda-setting theory. Social media data, such as the Twitter data discussed in this paper, reveal richer information about people’s reactions to social events.

South Africa takes the central position on this issue. From the contents of tweets and sentiment distribution, it is found that most of the debate is related to South Africa, specifically over the legalization of rhino horn trade, and they show a strong negative attitude towards the rhino trade. In fact, the debate among experts on whether to legalize rhino trade or not, has never stopped in nearly 20 years. The literature points out that rhino trade is a complicated system influenced by the market demand and faced with uncertainty [42]. If poaching continues to increase without active suppression, it will lead to the South African rhinos’ extinction within the following two decades [63].

Despite people’s high expectations about social media being a platform to express their opinions, it is revealed that the extent to which a message poster can be heard largely depends on the social structure [64]; this has been found by analyzing influential users by mentioning pairs. In our study, institutional users, including many worldwide government organizations, non-governmental organizations, and media, are found to play key roles in the rhino trade issue. In addition, some individual companies focusing on providing platforms for people to discover, support, and organize campaigns around issues that impact the world are the most influential ones among Twitter users. No personal accounts are regarded as influential, according to mentioner–mentionee network.

Similar findings of such kinds of public concerns were previously presented. While studying global climate change, researchers found that mass media coverage and the elite influenced public opinions to a great extent [65]. Comments and opinions of mainstream institutions with the majority of hyperlinks are easily spread out, and ordinary users’ voices are usually hard to be widely heard [66], which also implies that the perspectives toward the focused discussions lack diversity; in other words, several vital opinions are magnified by the public’s repetition. From another perspective, it is illustrated that the “two-step flow” model [67], which emphasizes the roles that opinion leaders play in information diffusion is explanatory. Thus, opinion leaders remain indispensable on the issue of the rhino trade based on Twitter.

Consequently, when it comes to rhino trade, including wildlife trade, the strong implications of such institutional users cannot be ignored. Governments should make full use of these platforms to appeal to the public to learn more about the situations that the wildlife are faced with.

Combined with personal information extracted from tweets, we can learn that celebrities have offline activities regarding rhino trade and draw considerable public attention. However, their online performance is not as stirring, thus no one is found to be an influential user in information diffusion.

Compared with traditional mass media, including radio, television, and newspapers, user-generated content from social media platforms produced by the Internet has many advantages, thereby enabling us to learn about the trends of public opinion and its information flow [64]. Twitter, rich in spatial and temporal information, is a useful distributed network of human sensors [54]. What is discovered by mining tweets is helpful in rapidly grasping the public attention and attitude on the issue, and according to the Agenda-setting theory, the key users should be utilized by the government to generate more awareness about rhino conservation.

## 5. Conclusions

Illegal wildlife trade is a global issue, which threatens biodiversity to a great extent. With the development of social media, people have gained access to an ideal public platform where they can express their opinions about such issues.

In this study, the rhino-trade-related tweets were analyzed to reveal the value of social media data in conservation research. It collected a decade’s worth of posted messages with the keywords “rhino trade” from Twitter. This study used text mining techniques, including named entity recognition, sentiment analysis, and social network analysis, to find out what interests people about this topic.

This study provides several important findings: (1) Through analyzing the relationship between online news and Tweet numbers, the tweeting behaviors are found to be affected by the traditional mass media releases. However, this paper also obtains rich information about public opinions through social media mining. (2) In general, countries and regions with strong negative sentiment tend to have a high volume of rhino trade cases, such as the United States and South Africa. However, sentiment analysis shows that not every country which has a high volume of trade cases displays concern about the illegal rhino trade. (3) From Tweet contents, social celebrities such as Prince William and Edna Molewa arouse wide public concern when they participate in conservation-related activities, but the influence lasts for a short duration, which is usually no- more than a month. Through social network analysis, this study discovered that the most influential users in this topic belong to the following four categories: NGOs, GOs, media, and individual enterprises.

It is revealed that this study can discover more useful information on the given topic from social media than it can from traditional media and can learn about public behaviors on social media platforms. This paper also studied the geographical distribution of public sentiment, noting that although the issue instigates wide concerns, certain key countries and regions show weak negative attitudes. According to the Agenda-setting theory, the key factors influencing public concern can be highlighted and utilized to enhance public awareness.

However, this study still has some limitations. First, in the data collecting phase, it chose only those tweets that are written in English. Other languages may also have been used in discussing the topic. Second, automated sentiment analysis by using NLP cannot completely reflect the true feelings of human beings.

To sum up, by analyzing social media data, governments and organizations may be advised to make full use of social media platforms to spread news and publicize celebrities’ activities about wildlife conservation. Particularly, in those countries with a high volume of trade but a low negative sentiment index, governments and influential organizations should take the initiative to arouse the public’s awareness about conservation by using social media platforms.

## Figures and Tables

**Table 1:** Trade cases of wild-sourced rhino for commercial, hunting, or personal purposes and its origins.

Origination	Number of Cases
ZA	857
NA	94
ZW	5
PT	3
AU	2
SZ	2
BW	1
DE	1
US	1
ID	1
IT	1
LT	1
ZM	1

**Table 2:** The destinations and the number of trade cases of wild-sourced rhino for commercial, hunting, or personal purposes from South Africa.

Destination	Number of Case
US	125
CN	54
RU	54
VN	50
ES	44
PL	42
DE	42
DK	37
UA	33
CZ	29
FR	29

**Table 3:** Profile of the users with outdegree over 100.

User Id	Outdegree	User Type
Change	1703	Private Company
Avaaz	1577	NGO
NRDC	1486	NGO
HSIGlobal	1196	NGO
NatGeo	704	Media
TakePart	633	Media
CITES	465	GO
WWF	439	NGO
USFWS	314	GO
EleRhinoMarch	273	Private Company
environmentza	273	GO
USFWSIntl	239	GO
guardian	221	Media
causes	200	Private Company
UKChange	180	Private Company
savetherhino	176	NGO
AWF_Official	175	NGO
ForceChange	155	Private Company
africageo	152	Media
sharethis	147	Media
CITESconvention	134	GO
NPR	131	Media
po_st	127	Media
USFWSHQ	126	GO
WildAid	122	NGO
c0nvey	120	Media
News24	116	Media

**Table 4:** Aggregated information of top users.

User Type	Total Outdegree	User Number	Average Outdegree per User
Media	2351	9	261
NGO	5171	7	739
GO	1551	6	259
Private Company	2511	5	502

**Table 5:** The rank of countries owning negative emotional index (top 5).

Year	Top 5 Negative Emotional Index				
2009	ZA	KE	GB	BE	CA
2010	ZA	SG	GB	FJ	ZW
2011	ZA	IE	KE	GB	US
2012	ZA	GB	KE	AU	CA
2013	GB	ZA	NZ	IE	AU
2014	ZA	GB	KE	AU	CA
2015	ZA	GB	KE	US	CA
2016	ZA	GB	KE	US	CA
2017	GB	ZA	US	AU	KE

## Data Availability

The rhino trade data can be found on CITES (https://trade.cites.org, accessed on 10 May 2022). The poaching stats can be found on SAVE THE RHINO (https://www.savetherhino.org/rhino-info/poaching-stats/, accessed on 10 May 2022).
